# Elucidation of new condition-dependent roles for fructose-1,6-bisphosphatase linked to cofactor balances

**DOI:** 10.1371/journal.pone.0177319

**Published:** 2017-05-25

**Authors:** Du Toit W. P. Schabort, Stephanus G. Kilian, James C. du Preez

**Affiliations:** Department of Microbial, Biochemical and Food Biotechnology, University of the Free State, Bloemfontein, South Africa; Universite Paris-Sud, FRANCE

## Abstract

The cofactor balances in metabolism is of paramount importance in the design of a metabolic engineering strategy and understanding the regulation of metabolism in general. ATP, NAD^+^ and NADP^+^ balances are central players linking the various fluxes in central metabolism as well as biomass formation. NADP^+^ is especially important in the metabolic engineering of yeasts for xylose fermentation, since NADPH is required by most yeasts in the initial step of xylose utilisation, including the fast-growing *Kluyveromyces marxianus*. In this simulation study of yeast metabolism, the complex interplay between these cofactors was investigated; in particular, how they may affect the possible roles of fructose-1,6-bisphosphatase, the pentose phosphate pathway, glycerol production and the pyruvate dehydrogenase bypass. Using flux balance analysis, it was found that the potential role of fructose-1,6-bisphosphatase was highly dependent on the cofactor specificity of the oxidative pentose phosphate pathway and on the carbon source. Additionally, the excessive production of ATP under certain conditions might be involved in some of the phenomena observed, which may have been overlooked to date. Based on these findings, a strategy is proposed for the metabolic engineering of a future xylose-fermenting yeast for biofuel production.

## Introduction

Recently, differential RNA-seq transcriptomics of *Kluyveromyces marxianus* was performed with glucose or xylose as the carbon source under aerobic conditions [1, 2]. It is to be expected that much of the differential response results from glucose derepression, as is the case with *Saccharomyces cerevisiae* in the absence of glucose, where the response is due to carbon source responsive transcription factors such as Adr1 and Mig1. Although the overall pattern of regulation in *K*. *marxianus* grown with D-xylose as carbon source instead of glucose resembled that of glucose derepression in *S*. *cerevisiae*, including the strong up-regulation of peroxisomal metabolism [1, 2], it did not represent a complete gluconeogenic response. For instance, the glyoxylate cycle was not up-regulated. Other signals are seemingly required for up-regulation of the glyoxylate cycle, which would be required if the cells were growing on acetyl-CoA arising from the β-oxidation of lipids. Some of the genes under glucose repression in *S*. *cerevisiae* have been dubbed “gluconeogenic” genes. Of particular interest is the fructose-1,6-bisphosphatase (FBP) reaction, catalysed by the Fbp1 protein and encoded by the FBP1 gene, which is under the control of the transcription factor Mig1 and glucose repression [3]. What distinguishes this gene from other genes subject to glucose repression is its centrality to energy metabolism. If the FBP1 gene is expressed, it is likely to have an effect on energy metabolism, whereas unused transporters and β-oxidation would not have an effect if the carbon source were glucose or xylose. It is thus possible that the FBP1 gene might take on a different role, not related to gluconeogenesis, under some derepressed conditions. The RNA-seq data on *K*. *marxianus* [2] revealed that the FBP1 gene was up-regulated 27-fold when xylose served as carbon source. Moreover, it was recently discovered that in each of more than six hundred tumours of clear cell renal cell carcinoma analysed in humans, the expression level of the FBP1 gene was decreased [4]. Hence, a deeper understanding of the regulation and dis-regulation of the FBP reaction may as well be important for the treatment of cancer.

The up-regulation of the FBP1 gene in a recombinant *S*. *cerevisiae* strain fermenting xylose has been postulated as a mechanism to increase NADPH production by a type of cyclic pentose phosphate pathway (PPP) [5]. NADPH is also required for xylose utilisation by a yeast such as *K*. *marxianus*, in which xylose reductase utilises NADPH for reducing power [6, 1]. However, the FBP reaction could also have a direct influence on ATP levels. In mammalian muscle cells, both the FBP and phosphofructokinase (PFK) reactions act simultaneously in a substrate cycle, effectively dissipating free energy by synthesising and hydrolysing ATP. This mechanism allows a greater dynamic range in regulation of the flux, making use of adenylate kinase and AMP as a signal amplifier mechanism for dynamic responses to changes in ATP concentration [7, 8]. In other cells the substrate cycle could also serve as a heat generation method, as in the case of the flight muscles of bumblebees [9]. Heat generation is, however, unlikely to be a physiological response in yeasts, as is the signal amplifier mechanism that facilitates the dynamic response in glycolytic flux in mammals.

Another type of non-shivering thermogenesis is present in mammals, in which brown fat tissue catabolises acetyl CoA from lipids to generate heat. To avoid excessive ATP production, or equivalently, to avoid a limitation in ADP, uncoupling mechanisms exist. Hence, ATP can be excessive and metabolism is not always geared towards producing the maximal yield of ATP from a substrate. In fact, the uncoupling proteins found in mammals are an elaborate mechanism to avoid excessive ATP production. These allow the pumping of protons across the mitochondrial membrane, generating heat in the process and avoiding the need for protons to pass through the F_1_F_0_ ATPase. Thermogenin (UCP1) and similar proteins in mammals are the primary uncoupling proteins that allow the passage of protons [10]. However, to date, dedicated uncoupling proteins have not been found in yeasts, and UPC1-like activity in the yeast *Yarrowia lipolytica* is due to the promiscuous activity of an anion carrier protein [11, 12].

A number of questions arise from the above observations: (*a*) Could an experimental condition or a genetic manipulation exist that would cause an ATP imbalance by excessive production of ATP (and thus an ADP limitation) in a yeast such as *K*. *marxianus*, or is there always a sufficient demand for ATP by processes such as biomass formation? (b) If such a situation indeed existed and considering that dedicated uncoupling proteins apparently are absent in yeasts, would substrate cycles like those induced by the FBP and PFK reactions be good candidate replacement mechanisms for uncoupling proteins under such conditions? (*c*) Further, since the FBP reaction is such a central reaction, with links to both ATP as well as to NADPH (as it is associated with gluconeogenesis), what is the true purpose of up-regulation of FBP in *K*. *marxianus* in a xylose medium when there is no apparent need for the FBP reaction? The transaldolase and transketolase reactions of the non-oxidative PPP, which catalyse the route of carbon entry from xylose, indeed produce fructose-6-phosphate which is upstream of FBP in the glycolytic pathway. (d) Finally, might there be as yet unidentified genetic manipulations which could theoretically enable anaerobic xylose fermentation for bioethanol production, given the complexities imposed by cofactor balances and flux constraints? These complex questions call for a rigid mathematical modelling framework such as Flux Balance Analysis (FBA). In this report, the complex interplay between cofactor balances, major metabolic pathways and redox cofactor specificity was investigated using FBA as a predictive simulation framework.

## Methods

The RNA-seq transcriptomic data were obtained as described elsewhere [2]. Briefly, *Kluyveromyces marxianus* strain UFS-2791 was cultivated at 35°C under aerobic conditions in a chemically defined medium containing glucose or xylose as the carbon source. The FBA model, capturing 56 reactions throughout central metabolism with reaction blocks for biomass formation, electron transport and oxidative phosphorylation, was described previously (see Schabort et al. [2], supplementary materials). The biomass formation formula was obtained from Fischer et al. [13] (See supplementary Table S5 in [2]). The phosphate/oxygen ratio (P/O ratio), which refers to the number of ADP to ATP conversions by ATP synthase per oxygen atom, was assumed to be 2.5 to simulate metabolism in Crabtree negative yeasts. The FBA simulation framework was described by Schilling et al. [14]. FBA was implemented in *Reactomica* using the Wolfram language. Flux constraints were defined by the stoichiometric matrix *S* and the exchange flux matrix *E* as below, which describe the mass balances of each metabolite as rows in the matrices.

Sv−Ee =0      (−∞≤v≤∞)

The intracellular flux vector v and the exchange flux vector e were calculated as a single vector, using optimisation with linear programming. In the above equation, *E* is an identity matrix that maps the vector of exchange fluxes to metabolite balances, where non-zero entries were only present for metabolites that can cross the cell boundary, or those which were allowed to be produced in excess, such as ATP. The metabolites and cofactors that were allowed to accumulate are indicated in the Results section. The upper bounds for all intracellular fluxes were left unconstrained, while irreversible reactions were constrained to a lower flux value of zero (See supplementary Table S5 in [2]). The uptake flux of the carbon source was constrained to a value of 10 mmol h^-1^ g biomass^-1^ specific flux. No substrate cycles, which could result in very high fluxes, were allowed. These were identified by Flux Variability Analysis [15], implemented also in *Reactomica*. The objective function was optimisation of the growth rate. Fluxes are interpretable in terms of their relative ratios compared to the molar uptake rate of the carbon source.

## Results

The results of the flux balance analyses of *K*. *marxianus* grown on glucose and xylose as respective carbon sources are presented below. For clarity, a visual guide to the reversibility of reactions and the names of reactions is provided as S1 Figs.

### Is an increase in NADPH production the likely role for up-regulation of FBP1 in the xylose medium?

**Fig 1:**
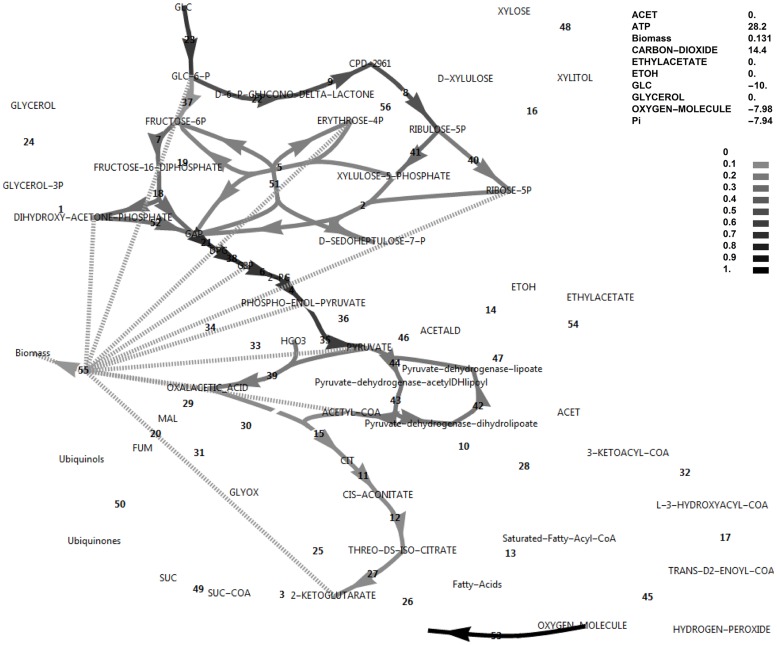
FBA simulation with glucose as *in silico* carbon source. Note that the glucose uptake flux was set to -10 and fluxes were interpreted relative to this flux as biomass specific fluxes, and that ATP is over-produced in this simulation at 28.2 as an exchange flux. This exchange flux represents reactions not directly accounted for in the model. Fluxes and exchange fluxes in these simulations are unitless, since they are determined by the upper bound of the carbon source uptake flux, set at a value of 10, and interpretation is done in the comparative sense. Reaction names, followed by MetaCyc ID’s are as follows (all maps of central metabolism contain the same annotations): 1, glycerol-3-phosphate dehydrogenase (NAD+) (1.1.1.8-RXN); 2, transketolase (1TRANSKETO-RXN); 3, 2-oxoglutarate dehydrogenase (2OXOGLUTARATEDEH-RXN); 4, phosphopyruvate hydratase (2PGADEHYDRAT-RXN); 5, D-fructose 6-phosphate:D-glyceraldehyde-3-phosphate glycolaldehyde transferase (2TRANSKETO-RXN); 6, phosphoglycerate mutase (3PGAREARR-RXN); 7, 6-phosphofructokinase (6PFRUCTPHOS-RXN); 8, phosphogluconate dehydrogenase (decarboxylating) (6PGLUCONDEHYDROG-RXN); 9, 6-phosphogluconolactonase (6PGLUCONOLACT-RXN); 10, acetate-CoA ligase (ACETATE—COA-LIGASE-RXN); 11, aconitate hydratase (ACONITATEDEHYDR-RXN); 12, aconitate hydratase (ACONITATEHYDR-RXN); 13, 2,3,4-saturated fatty acyl-CoA synthetase (ACYLCOASYN-RXN); 14, alcohol dehydrogenase (ALCOHOL-DEHYDROG-RXN); 15, citrate-S-synthase (CITSYN-RXN); 16, D-xylulose reductase (D-XYLULOSE-REDUCTASE-RXN); 17, enoyl-CoA hydratase (ENOYL-COA-HYDRAT-RXN); 18, fructose-bisphosphate aldolase (F16ALDOLASE-RXN); 19, fructose-bisphosphatase (F16BDEPHOS-RXN); 20, fumarate hydratase (FUMHYDR-RXN); 21, glyceraldehyde-3-phosphate dehydrogenase (phosphorylating) (GAPOXNPHOSPHN-RXN); 22, glucose-6-phosphate dehydrogenase (GLU6PDEHYDROG-RXN); 23, glucokinase (GLUCOKIN-RXN); 24, glycerol-1-phosphatase (GLYCEROL-1-PHOSPHATASE-RXN); 25, isocitrate lyase (ISOCIT-CLEAV-RXN); 26, isocitrate dehydrogenase (NADP^+^) (ISOCITDEH-RXN); 27, isocitrate dehydrogenase (NAD^+^) (ISOCITRATE-DEHYDROGENASE-NAD+-RXN); 28, acetyl-CoA-C-acyltransferase (KETOACYLCOATHIOL-RXN); 29, malate dehydrogenase (MALATE-DEH-RXN); 30, malate dehydrogenase (oxaloacetate-decarboxylating/malic enzyme) (NADP^+^) (MALIC-NADP-RXN); 31, malate synthase (MALSYN-RXN); 32, 3-hydroxyacyl-CoA dehydrogenase) (OHACYL-COA-DEHYDROG-RXN); 33, phosphoenolpyruvate carboxylase (PEPCARBOX-RXN); 34, phosphoenol pyruvate carboxykinase (ATP) (PEPCARBOXYKIN-RXN); 35, pyruvate kinase (PEPDEPHOS-RXN); 36, pyruvate, water dikinase (PEPSYNTH-RXN); 37, glucose-6-phosphate isomerase (PGLUCISOM-RXN); 38, phosphoglycerate kinase (PHOSGLYPHOS-RXN); 39, pyruvate carboxylase (PYRUVATE-CARBOXYLASE-RXN); 40, ribose-5-phosphate isomerase (RIB5PISOM-RXN); 41, ribulose-phosphate 3-epimerase (RIBULP3EPIM-RXN); 42, dihydrolipoyl dehydrogenase (RXN0-1132); 43, dihydrolipoyllysine-residue acetyltransferase (RXN0-1133); 44, pyruvate dehydrogenase (acetyl-transferring (RXN0-1134); 45, Acyl-CoA oxidase (RXN-11026); 46, pyruvate decarboxylase (RXN-6161); 47, acetaldehyde dehydrogenase (NAD^+^) (RXN66-3); 48, NADPH-dependent D-xylose reductase (RXN-8773); 49, succinate-CoA ligase (ADP-forming)) SUCCCOASYN-RXN); 50, succinate dehydrogenase (ubiquinone) (SUCCINATE-DEHYDROGENASE-UBIQUINONE-RXN); 51, transaldolase (TRANSALDOL-RXN); 52, triose-phosphate isomerase (TRIOSEPISOMERIZATION-RXN); 53, electron transport (vETC); 54, ethyl acetate synthesis (vEthylAcetate); 55, growth reaction (biomass formation) (vGrowth). 56, xylulokinase (XYLULOKIN-RXN).

**Fig 2:**
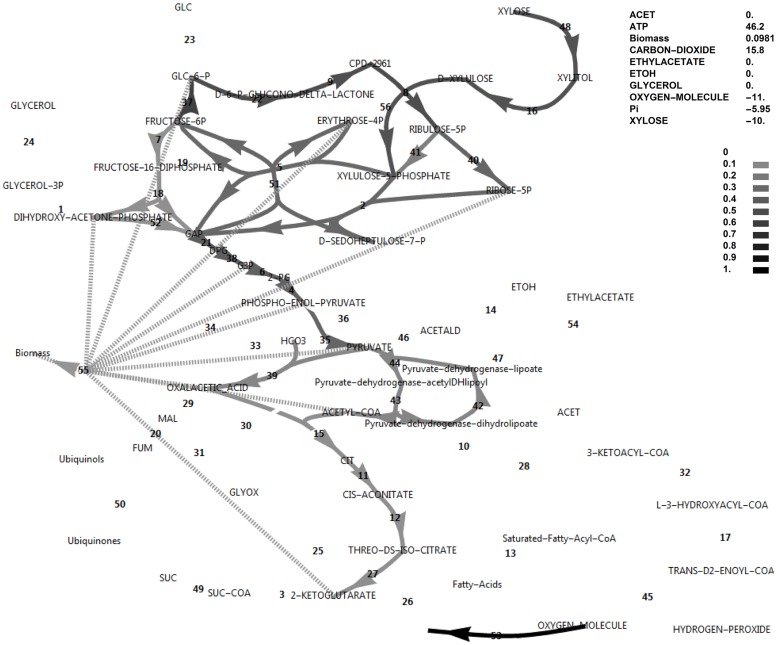
FBA simulation with xylose as *in silico* carbon source. Note that the xylose uptake flux was set to -10 and fluxes were interpreted relative to this flux as biomass specific fluxes, and that ATP is accumulated in this simulation at 46.2. The reaction names are as in Fig 1.

Simulations of aerobic metabolism in glucose and xylose media were initially performed. Figs 1 and 2 show the fluxes in the reference model in simulated glucose and xylose media, respectively. Note that for both simulations, the flux was limited by the same value for the sugar transporter at 10 mmol h^-1^ g^-1^, specific to cell dry weight. Although the experimental xylose uptake and growth rates were approximately 50% of those for glucose, the power of FBA lies in calculating fluxes relative to a reference flux; in this case, the sugar uptake flux. Also, the measured production rates of ethanol and acetate were not included here as hard constraints, to facilitate exploring the theoretical limits of the model. The biomass formation rate in these simulations have arbitrary units and should be treated in a comparative manner among conditions. Throughout, fluxes and exchange rates were interpreted in a comparative sense and the units of mmol h^-1^ g^-1^ were omitted.

Note that the reversible glucose-6-phosphate isomerase reaction flux switches direction between the conditions of glucose and xylose utilisation under simulated aerobic conditions (Figs 1 and 2). These simulations revealed that, although glucose-6-phosphate isomerase had to operate in the gluconeogenic direction when using xylose as an *in silico* carbon source, this was not the case for the PFK/FBP step and the FBP reaction did not become active when switching to the xylose *in silico* medium, in contrast to suggestions from literature that the FBP reaction was required to produce additional NADPH when xylose was the carbon source [5].

**Fig 3:**
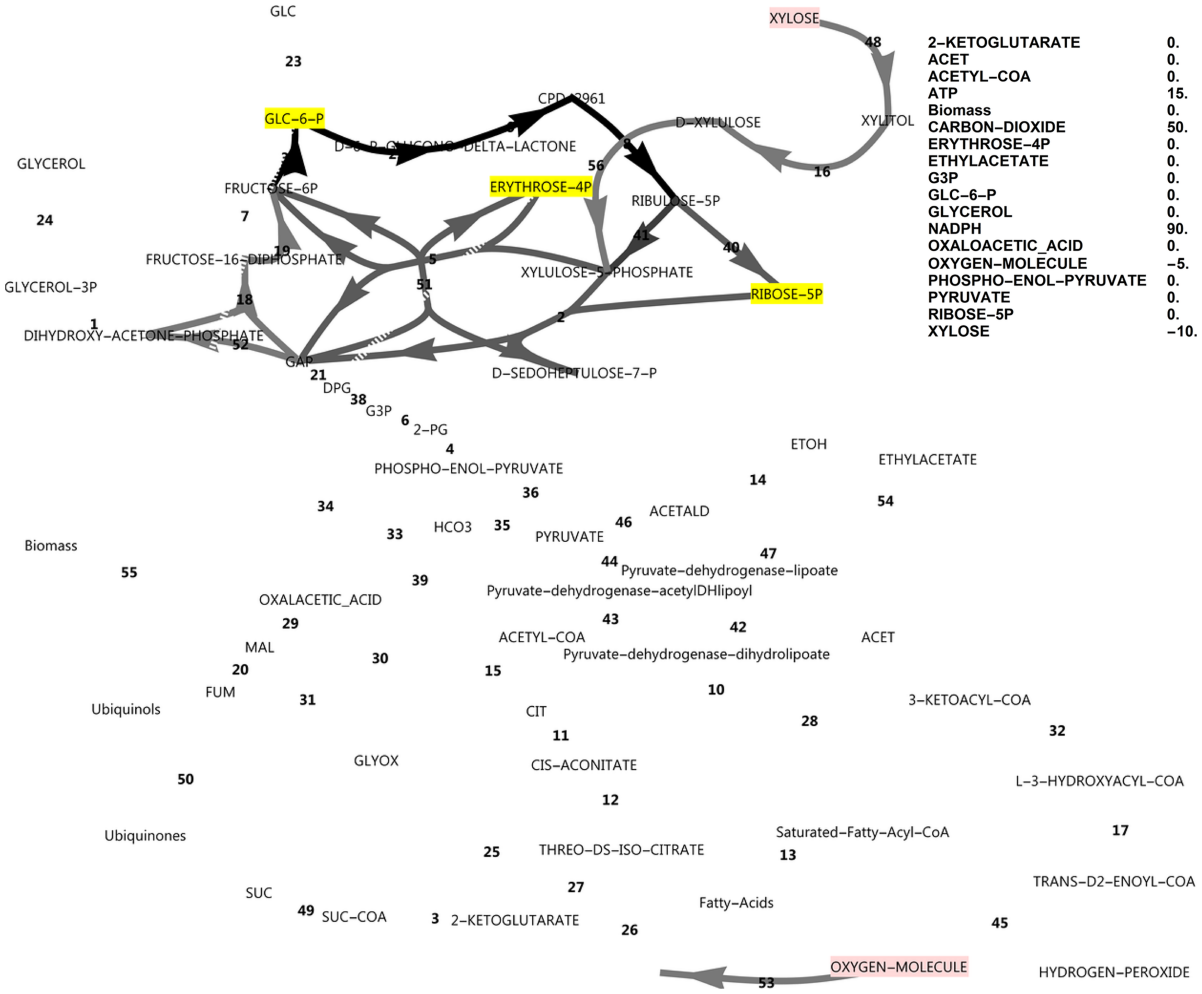
FBA simulation with xylose as *in silico* carbon source, inducing complete PPP cycling. Cofactors were allowed to exchange with the environment and FBP was active. Production of NADPH was optimised and complete cycling did not require glyceraldehyde-3-phosphate to accumulate. Nodes in pink indicate consumption and nodes in blue indicate production. Greyscale colours represent fluxes as a fraction of the highest flux in the simulation (black). None of the metabolites that were allowed to accumulate did accumulate (all yellow). The reaction names are as in Fig 1.

**Fig 4:**
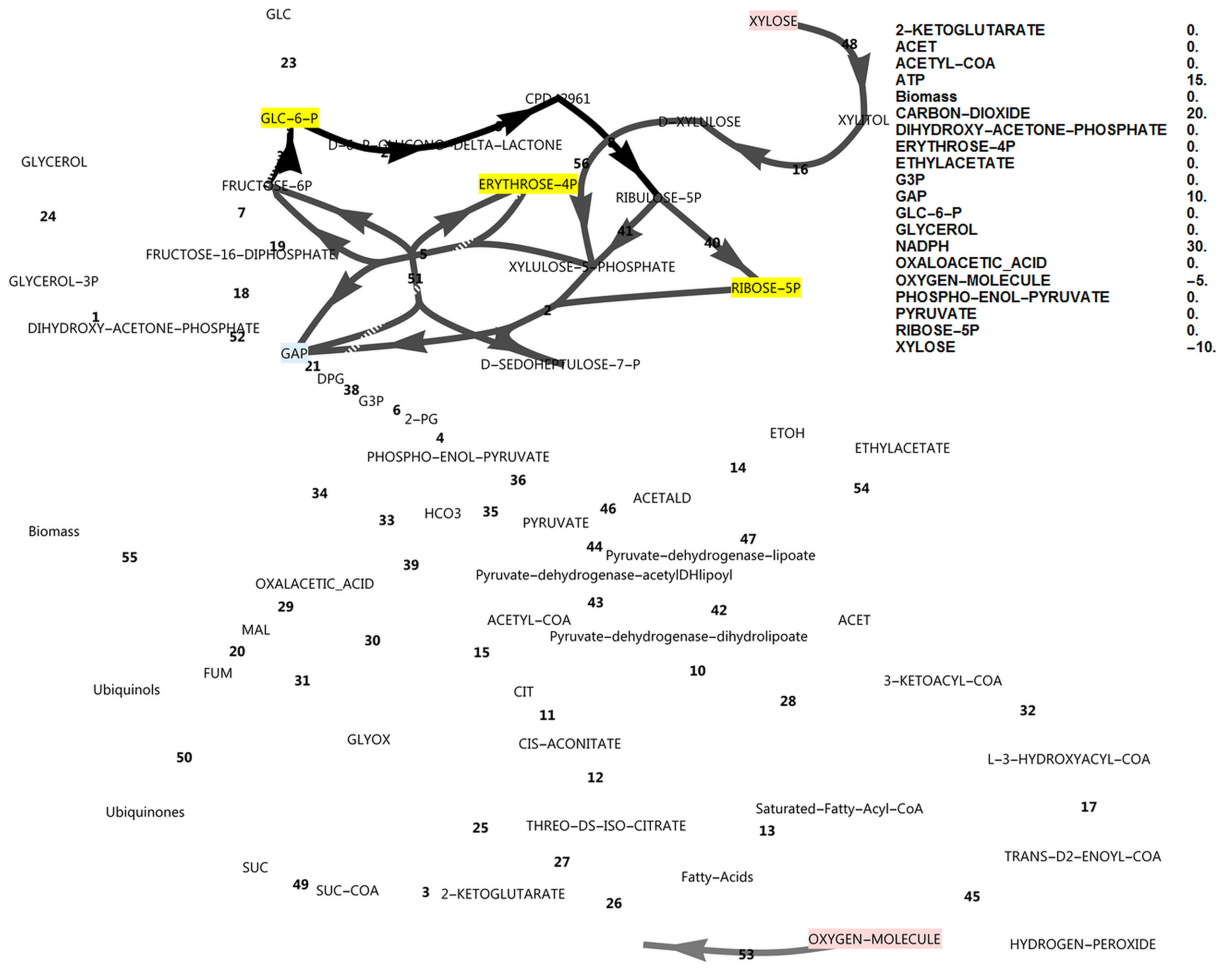
FBA simulation with xylose as *in silico* carbon source, inducing incomplete PPP cycling. Cofactor balances were open and FBP was inactive. Production of both NADPH and glyceraldehyde-3-phosphate were optimised. Nodes in pink indicate consumption, nodes in blue indicate production and nodes in yellow were allowed to accumulate, but did not accumulate. Greyscale colours represent fluxes as a fraction of the highest flux in the simulation (black). The reaction names are as in Fig 1.

By optimising for excessive unbalanced NADPH production from xylose, instead of the growth rate, it was found that, in principle, two modes of cyclic PPP flux were possible (Figs 3 and 4). Activating the FBP reaction (Fig 3) resulted in three-fold the molar yield of NADPH on substrate compared to the model without it (Fig 4), with an NADPH balance of 90 versus 30.

**Fig 5:**
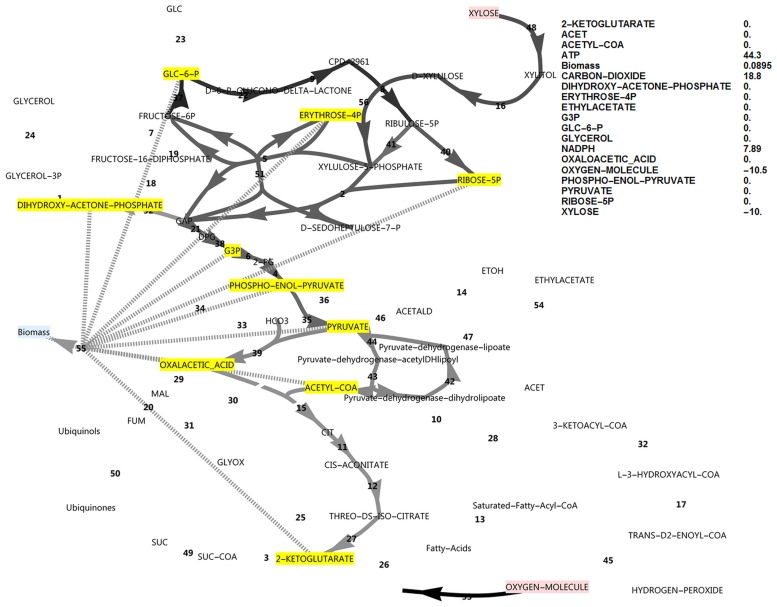
FBA simulation with xylose as the *in silico* carbon source, allowing overproduction of all biomass precursors as well as ATP and NADPH, with PFK inactive and FBP active. Note the absence of FBP flux and the substantial overproduction of only NADPH. Nodes in pink indicate consumption and nodes in yellow were allowed to accumulate, but did not accumulate. The reaction names are as in Fig 1.

However, when optimising for biomass production with a closed NADPH balance while both the unidirectional FBP and PFK reactions were activated, the flux was always glycolytic via PFK from glucose-6-phosphate to fructose-1,6-bisphosphate, with no flux through FBP. Deactivating PFK and activating FBP (attempting to force a complete cyclic PPP flux, with a gluconeogenic direction) allowed steady state only when NADPH was allowed to accumulate in the model (Fig 5) concomitant with a minor decrease in growth rate (0.0895 vs 0.0981), but FBP still did not carry flux. Null mutants of the PFK1 and PFK2 genes could thus be expected to have excessive reducing power in the form of NADPH when utilising xylose. This would manifest *in vivo* as a limitation in NADP^+^ regeneration from NADPH. The NADPH imbalance would be further increased in a scenario of a ‘gluconeogenic’ net flux via aldolase and FBP towards glucose-6-phosphate, which produces three-fold more NADPH per xylose molecule (shown in Fig 3). Thus, in this model where two molecules of NADPH are produced via the oxidative PPP, up-regulation of the FBP1 gene cannot result in a higher growth rate by supplying more NADPH, but in fact has the opposite effect.

**Fig 6:**
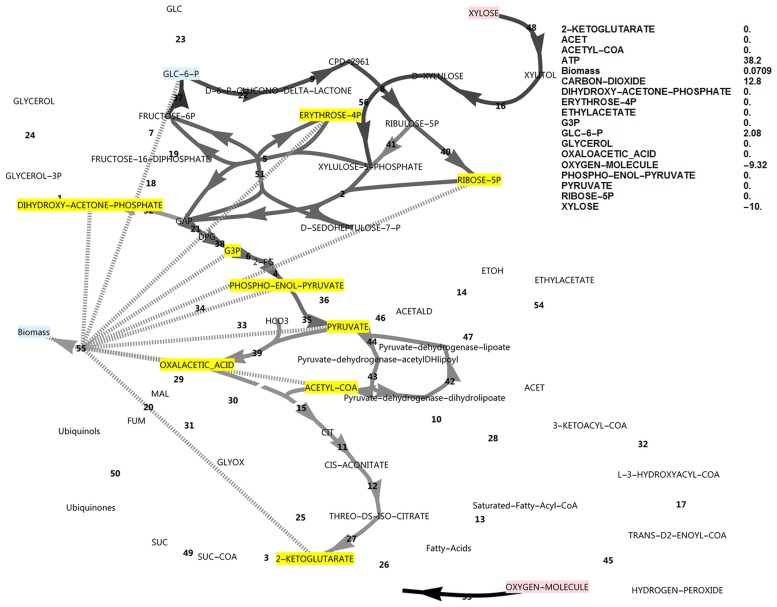
FBA simulation with xylose as *in silico* carbon source allowing overproduction of all biomass precursors as well as ATP, while closing the NADPH balance. PFK was inactive and FBP was active. Note the absence of FBP flux and the overproduction of glucose-6-phosphate. The reaction names are as in Fig 1.

An interesting observation was that intermediates such as glucose-6-phosphate would accumulate if the NADPH balance was closed and if both PFK and FBP were inactivated (Fig 6). The growth rate obtained in this scenario was somewhat lower than when the NADPH balance was open (0.0709 vs 0.0895). Thus, null mutants of the PFK1 and PFK2 genes may likely accumulate an intermediate such as glucose-6-phosphate during xylose utilisation, resulting in an overproduction of cell wall components, trehalose or glycogen. This finding could have an interesting biotechnological application. Nevertheless, this observation demonstrates that up-regulation of FBP in the absence of PFK cannot lead to a more balanced NADPH redox state and consequently a higher growth rate, as it would lead to a further excess of NADPH which may manifest as an accumulation of glucose-6-phosphate.

A cell may respond the constraint of NADPH overproduction, or NADP^+^ limitation, in one of several ways. Transdehydrogenases that oxidise NADPH while reducing NAD^+^ would be one such mechanism, but transdehydrogenases are absent in yeasts in general, except for *Pichia angusta* [16]. Conversely, trans-oxidation-reduction reaction cycles may occur in which NADPH is in effect exchanged for NADH in a cyclic pathway, might occur. Also, the cofactor preference of the oxidative PPP for NADP^+^ or NAD^+^ would affect the NADPH balance and, therefore, the potential role of FBP. The cofactor preference of oxidative PPP enzymes is known to vary among yeast species. The effect of cofactor preference in the oxidative PPP and the possibility of trans-oxidation-reduction reaction cycles in other parts of metabolism are explored in a later section. However, tightly linked to the redox cofactor balances is the ATP balance. These are linked not only via the oxidative metabolism in mitochondria, but also via the flux constraints in central metabolism, in which a given pathway may produce or consume ATP, NADH, NADPH and their partners in various ratios. Given that the total uptake rate of the carbon flux is constant in these simulations, alternative catabolic pathways with different cofactor stoichiometry in effect could cause a negative correlation between the production rates of different cofactors. Focussing still on upper central metabolism and the central role of FBP, the role of the ATP balance in flux routing is explored next.

### The ATP excess hypothesis and new roles for the FBP reaction and glycerol production

**Fig 7:**
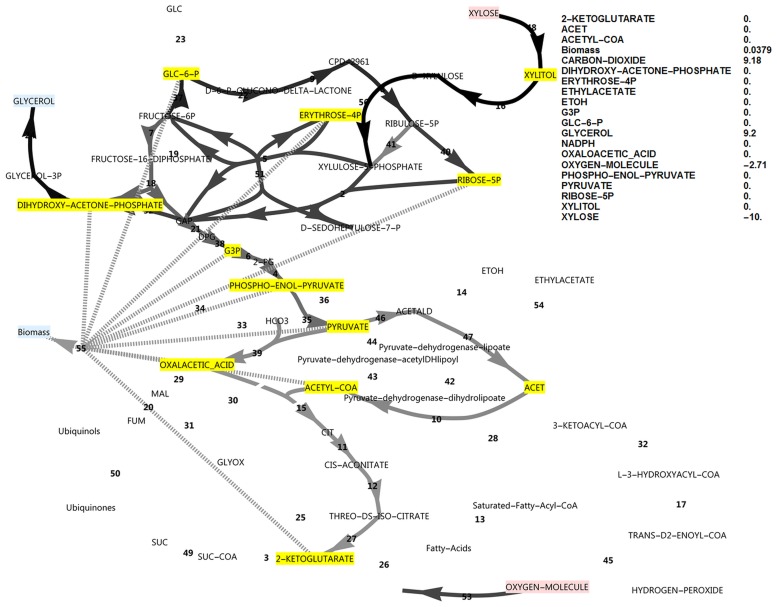
FBA simulation with xylose as *in silico* carbon source, allowing overproduction of all biomass precursors as well as NADPH, while closing the ATP balance. FBP activity was inactive while PFK was active. Note the production of glycerol and the appearance of flux in the pyruvate dehydrogenase bypass via acetate. The reaction names are as in Fig 1.

In the simulations discussed above, the ATP balance was not closed and ATP was allowed to accumulate. This exchange flux of ATP represents reactions that hydrolyse ATP and that are not included in the growth reaction and not accounted for in the modelled system. These may include the energy requirement for the synthesis and degradation cycles of biopolymers, the action of active transporters or unidentified metabolic substrate cycles. On both substrates a substantial positive ATP exchange flux was calculated, but notably the exchange flux of ATP was 64% higher in the xylose *in silico* medium compared to the glucose *in silico* medium (46.2 vs 28.2, Figs 1 and 2). Using xylose as carbon source, ATP supply was thus less likely to be growth rate limiting than when glucose was the carbon source, which is counter-intuitive. Also note that if the sugar transporter was to be changed to an active transporter, the effect would be negligible under these high ATP-yielding aerobic simulated conditions. Notably, it was found that closing the ATP balance caused a drastically decreased growth rate (0.0379 vs 0.0983), accompanied by both glycerol production and a flux through the pyruvate dehydrogenase bypass (PDB) (Fig 7). In this regard, glycerol production is a strategy to avoid NADH production in lower glycolysis and subsequent ATP synthesis resulting from electron transport, and is not due to a limited electron acceptor activity for regenerating NAD^+^, as is observed in *S*. *cerevisiae* under conditions supporting anaerobic growth [17]. The PDB hydrolyses two ATP equivalents through the acetate-CoA ligase step, whereas aldehyde dehydrogenase may produce one of either NADH or NADPH. Activation of the PDB led to a small increase in the growth rate but was non-essential for *in silico* growth, whereas glycerol production was essential.

This state thus resembles phenotypically, an oxygen limited growing phenotype which produces glycerol, involving a low oxygen consumption rate (-2.71). This effect is however induced as an ATP avoidance strategy. *In vivo*, it would manifest as a limitation in ADP, which may have its effect via enzyme kinetics on various enzymes.

Dedicated mechanisms in the electron transport chain for uncoupling ATP synthesis in yeasts have not been reported to date. Therefore, other mechanisms may be required to deal with the higher relative ATP production from xylose. The FBP reaction may perform exactly such a function by inducing an ATP hydrolysing substrate cycle together with PFK. Like the other glycolytic enzymes, the PFK1 and PFK2 genes were down-regulated in the xylose medium (Table 1). Notably, while the two subunits of PFK1 and PFK2 were down-regulated from 1640.1 and 1856.4 FPKM (fragments per kilobase per million reads) to 341.0 and 395.4 FPKM, respectively, FBP1 was up-regulated from 13.7 to 374.6 FPKM. Thus, the mRNA levels of the PFK and FBP genes were brought from extremely different levels to very similar levels. The presence of highly similar expression levels of genes encoding enzymes has indeed been observed in cases of confirmed substrate cycling, such as in mammalian muscle cells and bumblebees [7, 9]. Also, based on the striking similarity between simulated flux patterns and RNA-seq levels of genes in central metabolism [2], it seems that such deductions could be made in a pragmatic sense, linking the accurate transcript abundance levels from RNA-seq to protein levels and, ultimately, to an approximation of fluxes in the comparative sense.

**Table 1:** Relative expression levels (in FPKM) of mRNA in glucose and xylose media as determined by RNA-seq.

Gene name	Glucose	Xylose	Fold change from glucose to xylose
PFK1	1640.1	341	0.21
PFK2	1856.4	395.4	0.21
FBP1	13.7	374.6	27.34

**Fig 8:**
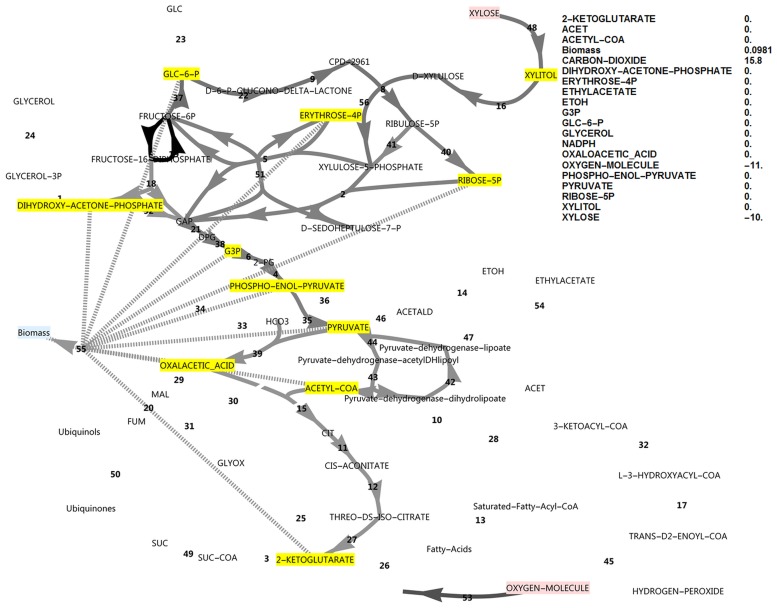
FBA simulation with xylose as *in silico* carbon source allowing overproduction of all biomass precursors as well as NADPH, while closing the ATP balance and with both FBP and PFK reactions active. Note the high growth rate, the absence of glycerol production and pyruvate dehydrogenase bypass fluxes, and the FBP/PFK substrate cycle that is responsible for the balance in ATP and ADP. The reaction names are as in Fig 1. The PFK/FBP substrate cycle serves as an alternative ATP sink in the absence of the ATP exchange flux, which represents various mechanisms of ATP utilisation.

Activating both the FBP and PFK activities and balancing ATP restored the rapid growth rate and avoided glycerol formation (Fig 8). The PFK/FBP substrate cycle induced here served as an alternative ATP sink in the absence of the ATP overproduction flux, representing ATP uncoupling, ATPases, or ATP-dependent membrane transporters. In this situation, activation and de-activation of the PDB made no difference to the growth rate or metabolite balances. The differential transcriptomic response of an engineered *S*. *cerevisiae* strain to xylose under anaerobic conditions was recently determined, which also showed a four-fold up-regulation of FBP1 in the presence of xylose [18]. Details of this substrate cycle can be seen in S1 Figs.

### Effect of cofactor specificity of oxidative PPP enzymes

**Fig 9:**
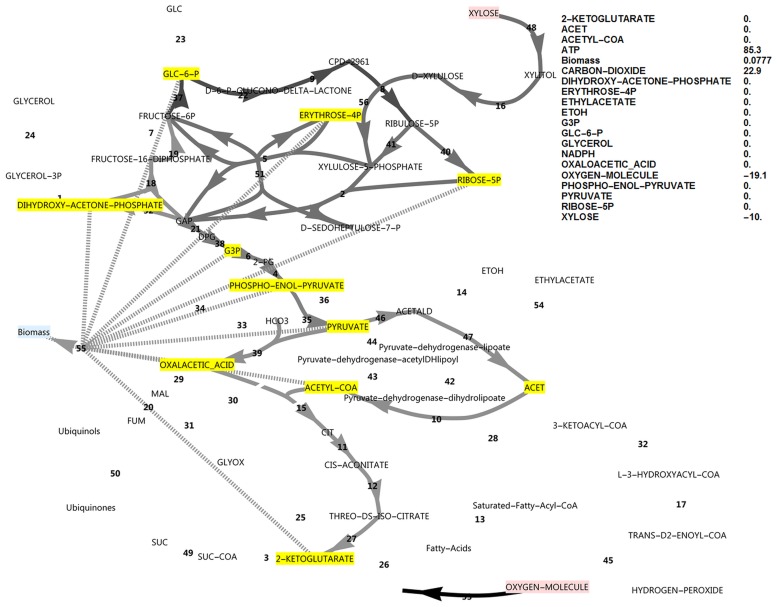
FBA simulation with xylose as *in silico* carbon source, assuming that the oxidative PPP produces one NADPH and one NADH. All biomass precursors as well as NADPH and ATP were allowed to accumulate and with FBP active and PFK inactive. Note the complete cyclic PPP flux, a large ATP exchange flux and the PDB flux. The reaction names are as in Fig 1.

**Fig 10:**
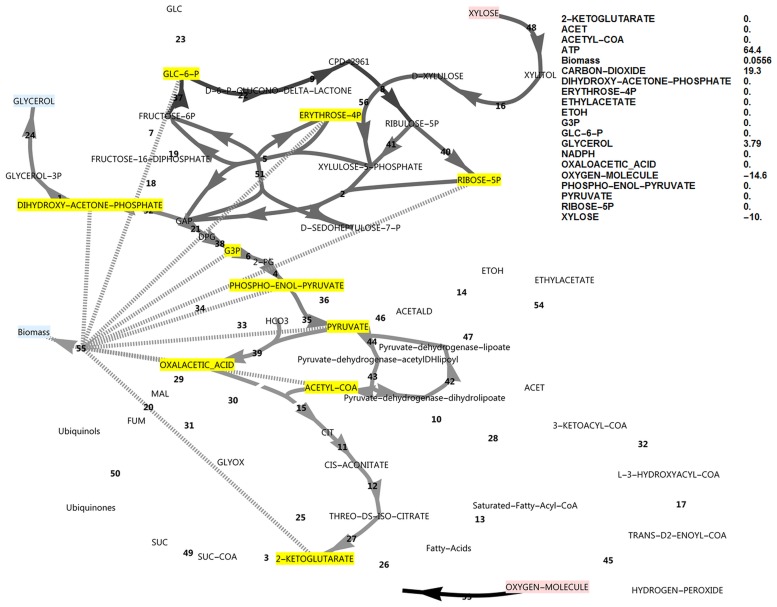
FBA simulation with xylose as *in silico* carbon source, assuming that the oxidative PPP produces one NADPH and one NADH. All biomass precursors as well as NADPH and ATP were allowed to accumulate and with PFK active and FBP inactive. Note the presence of glycerol production, the absence of PFK flux and a large ATP exchange flux. The growth rate was lower compared to the model in Fig 9 where FBP was active. The reaction names are as in Fig 1.

Although data on the subject of redox cofactor specificity is scarce, it has been found that in a number of bacteria [19, 20], the oxidative PPP enzymes such as glucose-6-phosphate dehydrogenase are not exclusively specific to NADP^+^, but can also use NAD^+^. In an attempt to obtain an increased cyclic PPP flux in the model, the cofactor specificity of glucose-6-phosphate dehydrogenase was changed in the model from NADP^+^ to NAD^+^ while allowing all biomass precursors and ATP to accumulate. As there was a deficiency of NADPH production in this model, cyclic PPP flux through FBP was observed as well as a higher growth rate (0.0777, Fig 9) than when FBP was absent (0.0556, Fig 10). A further increase in ATP exchange flux was present in this cyclic PPP mode (85.3, Fig 9), which was nearly double that of the reference model with a glucose-6-phosphate dehydrogenase specificity for NADP^+^ (46.2, Fig 2), and three-fold that of the initial model with glucose as carbon source (28.2, Fig 1). Thus, by allowing oxidative PPP enzymes to utilise NAD^+^ instead of NADP^+^, the FBP reaction may adopt a dual role. It would allow increased production of NADPH by the oxidative PPP, but at the same time this cyclic flux induces an increased overproduction of ATP, requiring the presence of the FBP/PFK substrate cycle—the second role of the FBP reaction. Absence of both FBP and PFK results in glycerol production, a lower growth rate and a substantial ATP overproduction (Fig 10). Although PDB can hydrolyse some of the excessive ATP, it cannot be compared to the potential of a substrate cycle to hydrolyse ATP, as it cannot carry a flux higher than 50% of the flux in lower glycolysis.

### Simulated anaerobic conditions

**Fig 11:**
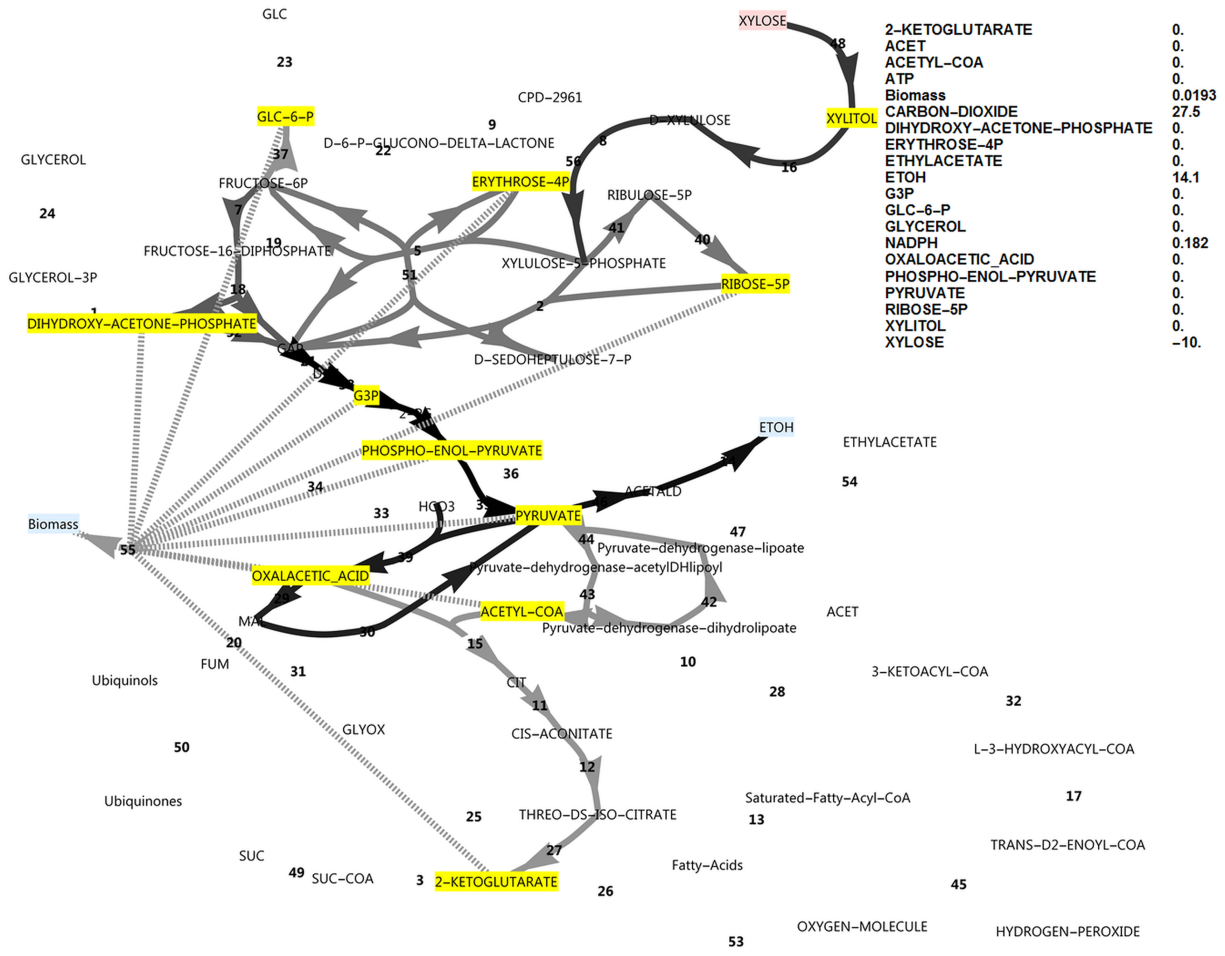
FBA simulation with xylose as *in silico* carbon source under anaerobic conditions. All biomass precursors as well as NADPH and ATP were allowed to accumulate and with both FBP and PFK active. Note the presence of ethanol production, absence of FBP flux or ATP accumulation, and the presence of a cyclic flux involving malic enzyme (reaction 30) which was required for *in silico* growth. Limiting malic enzyme activity caused the accumulation of xylitol. The reaction names are as in Fig 1.

**Fig 12:**
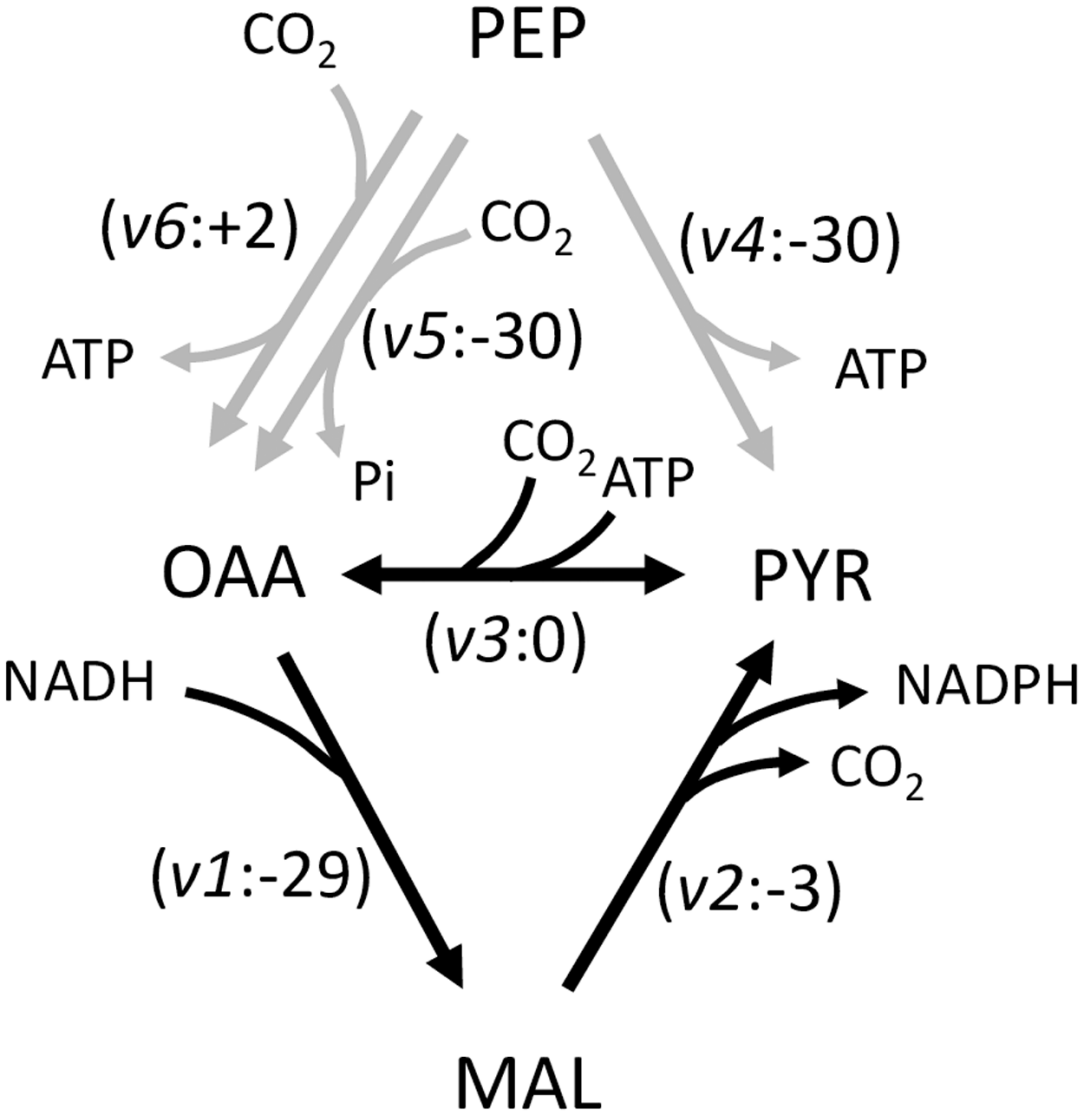
Reactions in anaplerosis that may enable a malic enzyme cycle. Reactions in black indicate the cycle. Reaction names are as follows: *v1*, malate dehydrogenase; *v2*, malic enzyme (NADP^+^-dependent/decarboxylating malate dehydrogenase); *v3*, pyruvate carboxylase; *v4*, pyruvate kinase; *v5*, phosphoenolpyruvate carboxylase; *v6*, phosphoenolpyruvate carboxykinase. OAA: oxaloacetate. PYR: pyruvate. MAL: malate. PEP: phosphoenolpyruvate. Pi: orthophosphate.

The FBA model, which is independent from the RNA-seq data, also provides a means to explore the theoretical potential of future engineered yeast strains. From this perspective, metabolism is only limited by the flux constraints, the activity bounds assumed for the uptake rate of the carbon source, and thermodynamic feasibility of the individual reactions, and assumes that gene expression levels can be suitably set by the metabolic engineer. In contrast to the aerobic scenario, under simulated anaerobic conditions (Fig 11) ATP production was balanced with NADPH approximately balanced. As a small overproduction of NADPH occurred, additional flux through the cyclic oxidative PPP was not required and thus neither was FBP required for additional NADPH production nor for ADP regeneration. Flux through glucose-6-phosphate isomerase, functioning in the gluconeogenic direction, was in the model only for the formation of cell wall glucans. Most important, however, was that oxaloacetate-decarboxylating (NADP^+^-requiring) malic enzyme activity was required to be active in order to obtain a steady state in these simulations. Together with pyruvate carboxylase and malate dehydrogenase of the TCA cycle, these enzymes formed a substrate cycle in the simulation that effectively oxidised the excess NADH, originating from xylulose reductase activity, to NAD^+^ and supplied additional NADPH for xylose reductase. At the same time, excess ATP was consumed by the cycle, which resulted in the balancing of ATP production and utilisation. These reactions are also shown in Fig 12. Limiting the capacity of malic enzyme *in silico* also manifested as xylitol accumulation, a phenomenon often observed in yeasts growing on xylose.

The gene for malic enzyme, MAE1, was found in our annotation against UniProt, and was constitutively expressed under the aerobic conditions tested in glucose and xylose media using RNA-seq [2]. To our knowledge, xylose fermentation by *K*. *marxianus* under anaerobic or oxygen-limited conditions has not been described thus far, which might be the relevant condition for the proposed malic enzyme cycle. It is also important to note that no flux was predicted in the oxidative PPP flux under the anaerobic condition utilising xylose. Sufficient NADPH for growth was produced by the oxaloacetate-decarboxylating malic enzyme. Ribose-5-phosphate was derived from xylulose-5-phosphate via ribulose-5-phosphate. PDB, which reportedly is utilised under anaerobic conditions [21], only contributed a minor effect to the growth rate, as an *in silico* knock-out of the PDB enzyme only resulted in a minor decrease in growth rate. From a flux perspective, its role is thus not clear for anaerobic conditions.

Responses of an engineered *S*. *cerevisiae* strain to xylose under anaerobic conditions was recently determined [18], showing a 6.5-fold down-regulation of the MAE1 gene in a xylose medium. However, since *S*. *cerevisiae* did not evolve for fermenting xylose, the latter observation cannot be extrapolated to *K*. *marxianus*. There was, however, a four-fold up-regulation of FBP1 in the recombinant *S*. *cerevisiae* strain when fermenting xylose.

## Discussion

Cofactor balance is an important consideration in developing a metabolic engineering strategy. However, even without taking enzyme kinetics into account, the multiple reactions that involve NAD^+^, NADP^+^ and ATP, combined with a variable and uncertain cofactor specificity for redox cofactors, render central metabolism complex to understand. FBA as a framework was demonstrated here to provide insight into the implications of alternative cofactor specificity of oxidative PPP enzymes. The potential role of the FBP reaction in cofactor balance was explored and, at the same time, its possible involvement in the ATP balance by forming a substrate cycle with PFK. It was shown that FBP could indeed contribute to additional NADPH production during xylose utilisation by causing a cyclic PPP flux, but only in a scenario where the oxidative PPP enzymes were not exclusively specific for NADP^+^ and only under aerobic conditions. Additionally, it was shown that in an *in silico* xylose medium, the excess ATP production was substantially greater than on glucose. This effect was increased further when the cofactor specificity of oxidative PPP enzymes for NAD^+^ increased. Thus, FBP may have a dual function under aerobic conditions—both to increase NADPH production in yeasts with one NAD^+^ -specific oxidative PPP enzyme and to hydrolyse excessive ATP.

Considering metabolic engineering of a future xylose-fermenting, bioethanol-producing yeast, at least three main aspects would be critical for success. Firstly, anaerobic growth requires an additional fermentative route for oxidation of the additional NADH that is produced by xylitol reductase and which cannot stoichiometrically be oxidised by alcohol dehydrogenases, as there is a shortage of electron acceptors. Secondly, it has been shown in *S*. *cerevisiae* that the glycolytic flux is strongly coupled to the yield of ATP during catabolism [22]. For instance, by using recombinant active transporters for sugars instead of facilitated diffusion in *S*. *cerevisiae*, the ethanol yield on sugar was increased while the biomass yield was decreased [23]. Since under xylose utilisation there seems to be a further increase in ATP overproduction, negative feedback on glycolysis by a high ATP concentration might amplify this problem. Thirdly, for every xylose molecule utilised, one NADPH would need to be oxidised which would have to be regenerated from NADP^+^ in another pathway, which might be the oxidative PPP, putting further flux constraints on the system. Notably, it was shown in this report that a potential malic enzyme cycle involving oxaloacetate-decarboxylating, NADP^+^-dependent malic enzyme could theoretically fulfil all three roles, enabling xylose fermentation, which currently is not theoretically possible in *K*. *marxianus* and other natural yeasts that do not possess a xylose isomerase gene. The cycle would oxidise the excessive NADH that cannot be oxidised by alcohol dehydrogenase due to flux constraints. In doing so, it also provides additional NADPH for the xylose reductase step for xylose utilisation. Finally, it forms an additional ATP sink, which may further pull glycolytic flux forward to ethanol production. Thermodynamic feasibility of this cycle involving malic enzyme should to be calculated based on the concentrations of ATP, ADP, Pi, NADH, NAD^+^, NADPH and NADP^+^ for this species, which is currently unknown. In addition, the PFK/FBP cycle may constitute an additional ATP sink, if it was found that ATP was inhibiting glycolytic flux. This cycle might, in fact, already be active in xylose-utilising *K*. *marxianus*, since our RNA-seq data showed that the FBP1 gene was derepressed in the xylose medium [2].

Other unanticipated findings were brought to light. Aerobic glycerol production as a physiological strategy to avoid excessive ATP production was also proposed here. Glycerol production is often observed in yeast fermentations and has primarily been associated with oxygen-limited growth conditions, but here the simulation demonstrated a different mechanism. The role of the PDB was also investigated. It was shown that allowance for a PDB flux increased the simulated growth rate under aerobic conditions when ATP was not allowed to be over-produced, as it hydrolysed ATP in the ATP over-producing scenario of aerobic xylose utilisation. In the case when oxidative PPP enzymes were more specific for NAD^+^, PDB may also improve growth since it produces additional NADPH. However, the potential contribution of PDB to these effects was low, as this flux was constrained by the flux in lower glycolysis. In a scenario of a defined mineral medium where the organism has to synthesise all biomass components from a sugar as carbon and energy source, as was simulated here, the potential flux through the PDB is further decreased as anaplerotic reactions from lower glycolytic intermediates subtract from the potential flux to PDB to replenish oxaloacetate for amino acid synthesis.

Two additional potential ATP sinks exist in yeasts. The first is another cycle in anaplerosis in which pyruvate and phosphoenolpyruvate are interconverted and dissipate free energy by effectively hydrolysing ATP [24, 25]. PEP carboxylase was not found in the *K*. *marxianus* UFS-Y2791 annotation, whereas PEP carboxykinase was constitutively expressed at low levels, as was pyruvate carboxylase [2]. Therefore, there was thus no indication of such a cyclic pathway being active. Another is the H^+^-ATPase of the cell membrane [17, 26, 27] as well as the ABC drug efflux pumps of *S*. *cerevisiae* [28]. H^+^-ATPase exports protons that originated from the proton symport of nutrients, including NH_4_^+^. The ATP-hydrolysing capacity of the efflux pumps, however, results in the pumping of protons and NH_4_^+^, and hence cannot act as condition-independent replacements for uncoupling proteins.

## Conclusions

This work shows that cofactor balances should not be interpreted separately. Furthermore, it highlighted the importance of experimentally determining the cofactor specificity of oxidative PPP enzymes, as the prediction or calculation of fluxes will change, depending on these parameters. The ATP balance is highly relevant to metabolic engineering, since ATP not only has a negative feedback on glycolysis, but also a high ATP concentration might lead to excessive biomass formation, reducing the yield of a primary fermentation product such as ethanol. Thus, from a practical perspective, the proposed substrate cycle induced by FBP may be useful in decreasing the effective ATP yield on glucose, which may lead to an increase in ethanol production. This cycle might already be employed by natural *K*. *marxianus* strains, since the FBP1 gene was up-regulated in the glucose-free xylose medium, as observed in RNA-seq data. In addition, it was proposed here that a highly active malic enzyme cycle, which effectively exchanges redox equivalents from NADH to NADP^+^, would not only solve a redox imbalance under anaerobic, xylose fermenting conditions, but would draw the glycolytic flux forward due to forming an ATP sink. Finally, it is evident that ATP should not be thought of as always in demand. Depending on the condition, it might become excessive, especially in the case of pentose utilisation. The FBP/PFK substrate cycle and its regulation probably plays a key role in energy homeostasis, even in yeasts under selected conditions, which could determine phenotype switching. Alteration of the activity of the FBP/PFK substrate cycle and its effects on cofactor balances of ATP, NADH and NADPH may also be a mechanistic explanation for the recent discovery of the strong causative link between mutations affecting the FBP1 gene and renal cell carcinoma in humans.

## Supporting information

S1 Figs Supplementary figures. (PPTX)
