# Supplementary material for: Elucidation of new condition-dependent roles for fructose-1,6-bisphosphatase linked to cofactor balances
Source: PLoS One. 2017 May 25;12(5):e0177319. doi: 10.1371/journal.pone.0177319 (PMC5444679; doi:10.1371/journal.pone.0177319)
Supplement: S1 Figs — (PPTX) [file pone.0177319.s001.pptx]

## Slide 1
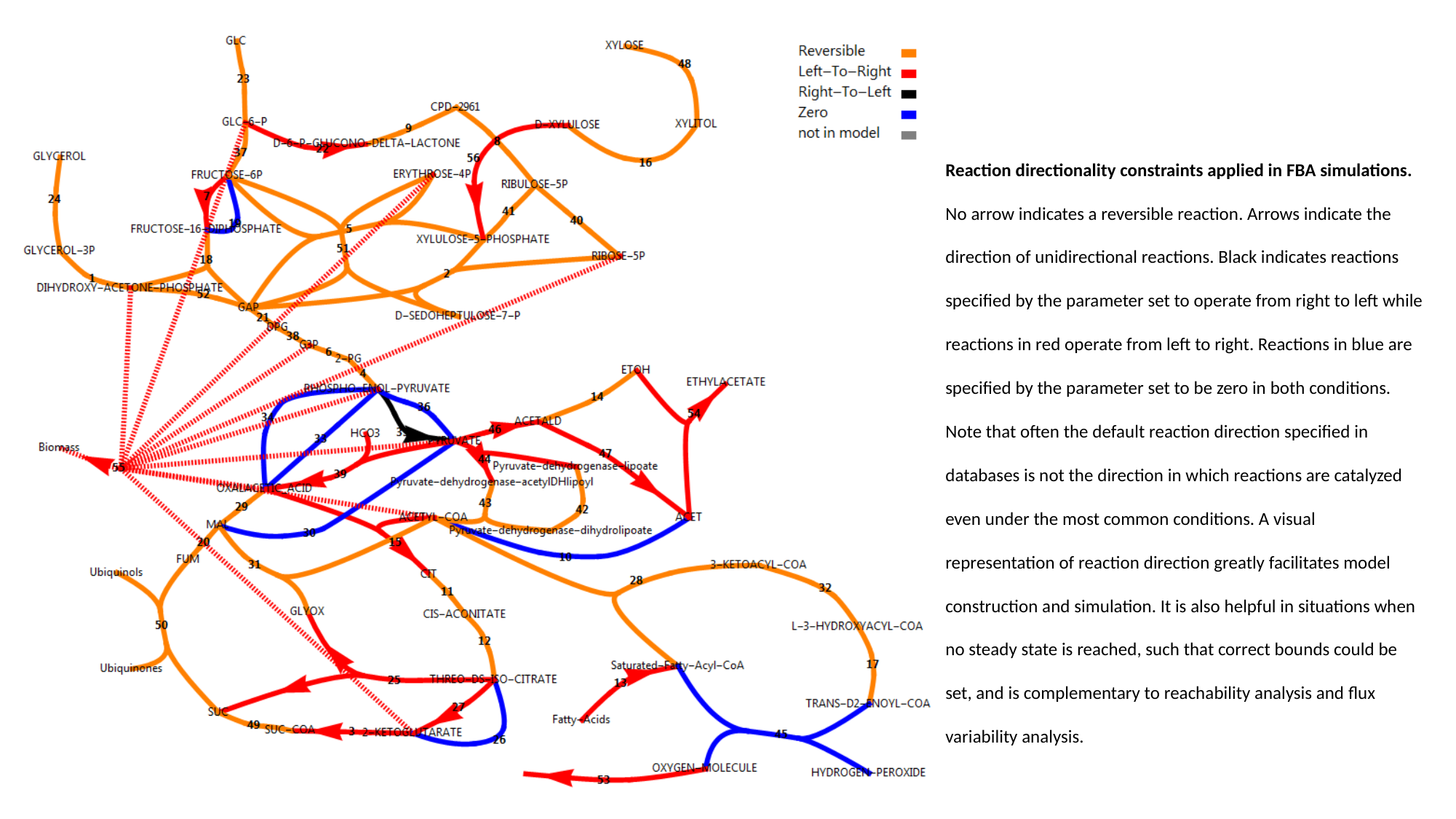

Reaction directionality constraints applied in FBA simulations. No arrow indicates a reversible reaction. Arrows indicate the direction of unidirectional reactions. Black indicates reactions specified by the parameter set to operate from right to left while reactions in red operate from left to right. Reactions in blue are specified by the parameter set to be zero in both conditions. Note that often the default reaction direction specified in databases is not the direction in which reactions are catalyzed even under the most common conditions. A visual representation of reaction direction greatly facilitates model construction and simulation. It is also helpful in situations when no steady state is reached, such that correct bounds could be set, and is complementary to reachability analysis and flux variability analysis.

## Slide 2
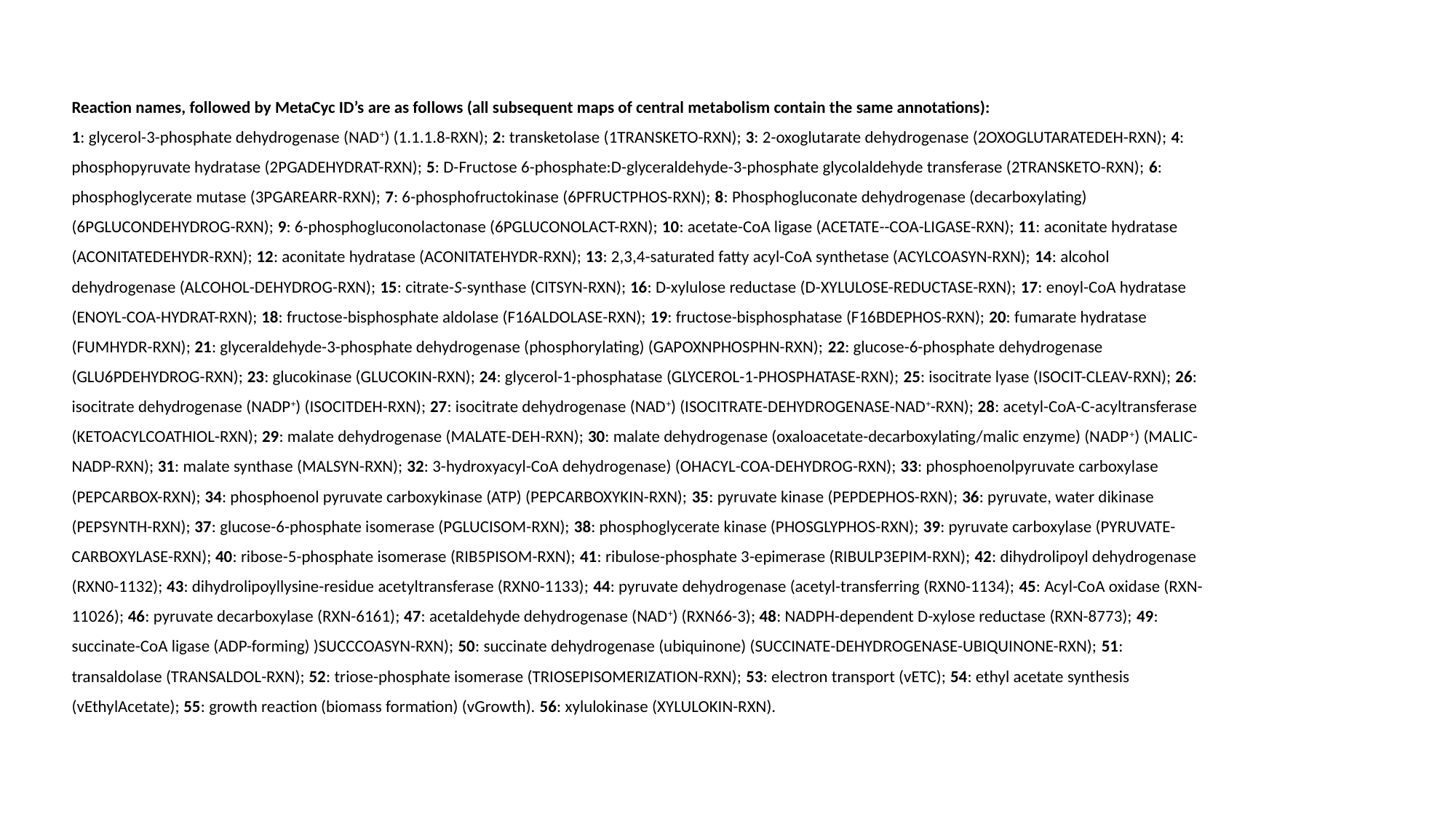

Reaction names, followed by MetaCyc ID’s are as follows (all subsequent maps of central metabolism contain the same annotations):
1: glycerol-3-phosphate dehydrogenase (NAD+) (1.1.1.8-RXN); 2: transketolase (1TRANSKETO-RXN); 3: 2-oxoglutarate dehydrogenase (2OXOGLUTARATEDEH-RXN); 4: phosphopyruvate hydratase (2PGADEHYDRAT-RXN); 5: D-Fructose 6-phosphate:D-glyceraldehyde-3-phosphate glycolaldehyde transferase (2TRANSKETO-RXN); 6: phosphoglycerate mutase (3PGAREARR-RXN); 7: 6-phosphofructokinase (6PFRUCTPHOS-RXN); 8: Phosphogluconate dehydrogenase (decarboxylating) (6PGLUCONDEHYDROG-RXN); 9: 6-phosphogluconolactonase (6PGLUCONOLACT-RXN); 10: acetate-CoA ligase (ACETATE--COA-LIGASE-RXN); 11: aconitate hydratase (ACONITATEDEHYDR-RXN); 12: aconitate hydratase (ACONITATEHYDR-RXN); 13: 2,3,4-saturated fatty acyl-CoA synthetase (ACYLCOASYN-RXN); 14: alcohol dehydrogenase (ALCOHOL-DEHYDROG-RXN); 15: citrate-S-synthase (CITSYN-RXN); 16: D-xylulose reductase (D-XYLULOSE-REDUCTASE-RXN); 17: enoyl-CoA hydratase (ENOYL-COA-HYDRAT-RXN); 18: fructose-bisphosphate aldolase (F16ALDOLASE-RXN); 19: fructose-bisphosphatase (F16BDEPHOS-RXN); 20: fumarate hydratase (FUMHYDR-RXN); 21: glyceraldehyde-3-phosphate dehydrogenase (phosphorylating) (GAPOXNPHOSPHN-RXN); 22: glucose-6-phosphate dehydrogenase (GLU6PDEHYDROG-RXN); 23: glucokinase (GLUCOKIN-RXN); 24: glycerol-1-phosphatase (GLYCEROL-1-PHOSPHATASE-RXN); 25: isocitrate lyase (ISOCIT-CLEAV-RXN); 26: isocitrate dehydrogenase (NADP+) (ISOCITDEH-RXN); 27: isocitrate dehydrogenase (NAD+) (ISOCITRATE-DEHYDROGENASE-NAD+-RXN); 28: acetyl-CoA-C-acyltransferase (KETOACYLCOATHIOL-RXN); 29: malate dehydrogenase (MALATE-DEH-RXN); 30: malate dehydrogenase (oxaloacetate-decarboxylating/malic enzyme) (NADP+) (MALIC-NADP-RXN); 31: malate synthase (MALSYN-RXN); 32: 3-hydroxyacyl-CoA dehydrogenase) (OHACYL-COA-DEHYDROG-RXN); 33: phosphoenolpyruvate carboxylase (PEPCARBOX-RXN); 34: phosphoenol pyruvate carboxykinase (ATP) (PEPCARBOXYKIN-RXN); 35: pyruvate kinase (PEPDEPHOS-RXN); 36: pyruvate, water dikinase (PEPSYNTH-RXN); 37: glucose-6-phosphate isomerase (PGLUCISOM-RXN); 38: phosphoglycerate kinase (PHOSGLYPHOS-RXN); 39: pyruvate carboxylase (PYRUVATE-CARBOXYLASE-RXN); 40: ribose-5-phosphate isomerase (RIB5PISOM-RXN); 41: ribulose-phosphate 3-epimerase (RIBULP3EPIM-RXN); 42: dihydrolipoyl dehydrogenase (RXN0-1132); 43: dihydrolipoyllysine-residue acetyltransferase (RXN0-1133); 44: pyruvate dehydrogenase (acetyl-transferring (RXN0-1134); 45: Acyl-CoA oxidase (RXN-11026); 46: pyruvate decarboxylase (RXN-6161); 47: acetaldehyde dehydrogenase (NAD+) (RXN66-3); 48: NADPH-dependent D-xylose reductase (RXN-8773); 49: succinate-CoA ligase (ADP-forming) )SUCCCOASYN-RXN); 50: succinate dehydrogenase (ubiquinone) (SUCCINATE-DEHYDROGENASE-UBIQUINONE-RXN); 51: transaldolase (TRANSALDOL-RXN); 52: triose-phosphate isomerase (TRIOSEPISOMERIZATION-RXN); 53: electron transport (vETC); 54: ethyl acetate synthesis (vEthylAcetate); 55: growth reaction (biomass formation) (vGrowth). 56: xylulokinase (XYLULOKIN-RXN).

## Slide 3
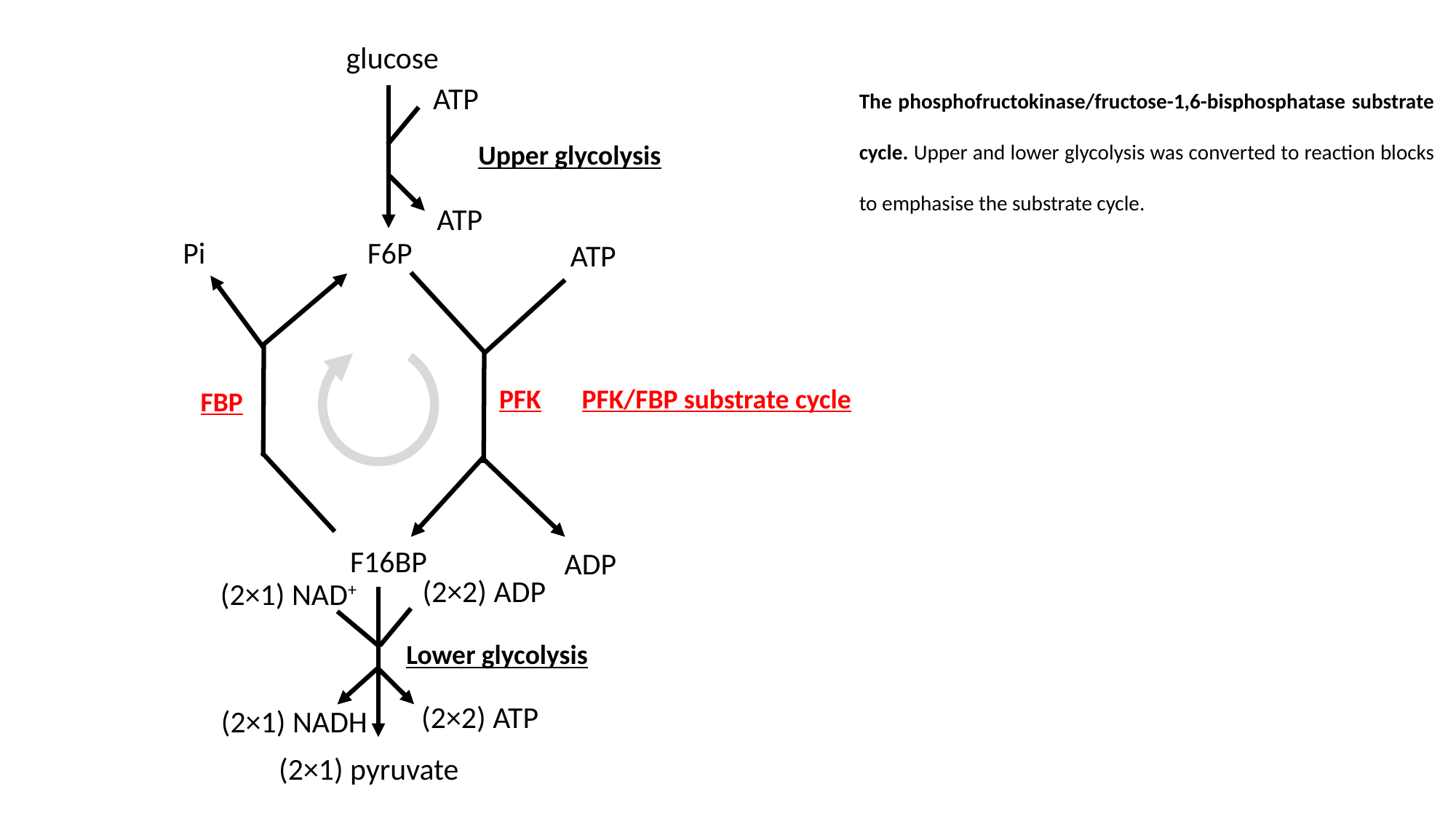

glucose
Upper glycolysis
Pi
F6P
ATP
PFK
F16BP
ADP
FBP
(2×1) NAD+
Lower glycolysis
(2×1) NADH
(2×1) pyruvate
The phosphofructokinase/fructose-1,6-bisphosphatase substrate cycle. Upper and lower glycolysis was converted to reaction blocks to emphasise the substrate cycle.
ATP
ATP
PFK/FBP substrate cycle
(2×2) ADP
(2×2) ATP
